# Perinatal depression and developmental risk of the infant: Analysis of a clinical sample and assessment of the impact of the COVID-19 pandemic

**DOI:** 10.1192/j.eurpsy.2021.278

**Published:** 2021-08-13

**Authors:** C. Pucci, M. Caccialupi O. P., M. Panfili, N. Giacchetti, F. Aceti, C. Sogos

**Affiliations:** Human Neuroscience, University of Rome - La Sapienza, Rome, Italy

**Keywords:** Mother-child interaction, Child development, COVID-19, Perinatal depression

## Abstract

**Introduction:**

Studies on large samples agree on the negative impact of maternal perinatal depression (PD) on child’s cognitive development. Early experience with insensitive maternal interactions appears to be predictive of poorer cognitive functioning. These children present a higher risk for the onset of socioemotional development, nutrition, growth and sleep disorders. Research on Covid-19 pandemic suggests that families, particularly mothers, may be at increased risk of psychological distress.

**Objectives:**

This study evaluates the effect of perinatal depression on child development and the impact of distress caused by the Covid-19 pandemic.

**Methods:**

We designed a case-control study comparing, during Covid-19 pandemic, a group-A of children of mothers with PD (n=19), with a group-B of children of healthy mothers (n=21). The age of the children recruited was 4-35 months. Participating mothers underwent DP3-Interview and the socioemotional and adaptive-behavior Bayley’s scales by telephone and completed an online survey (IES-R).

**Results:**

We found significantly lower scores on the Bayley socioemotional scale and in all the DP3-scales, in group-A. There is an inversely proportional correlation between the age of these children and overall development score of the DP3. On the IES-R scale, the medium scores in both groups show no psychological distress as a consequence of the Covid-19 pandemic, although mothers with PD show borderline total scores and higher hyperarousal scale values.

**Conclusions:**

This study confirms that PD is a risk factor for the onset of disorders in all areas of the child’s development. Mothers with PD are less likely to manage psychological distress secondary to the pandemic.

**Disclosure:**

No significant relationships.

